# Motivational Complexity of Nonsuicidal Self-injury Associated with its Severity in Emerging Adult University Students

**DOI:** 10.1007/s10964-025-02278-6

**Published:** 2025-10-31

**Authors:** Melinda Reinhardt, Gyöngyi Kökönyei

**Affiliations:** 1 Institute of Psychology, ELTE Eötvös Loránd University, Budapest, Hungary; 2 14th District Medical Center, Child and Adolescent Psychiatry, Budapest, Hungary; 3 NAP3.0-SE Neuropsychopharmacology Research Group, National Brain Research Program, Semmelweis University, Budapest, Hungary; 4 Department of Pharmacodynamics, Faculty of Pharmaceutical Sciences, Semmelweis University, Budapest, Hungary

**Keywords:** Nonsuicidal self-injury, Motivations, Severity, Latent profile analysis, Emerging adult university students

## Abstract

A comprehensive understanding of the functions of nonsuicidal self-injury (NSSI) requires an in-depth exploration of the diverse motivational patterns that underlie this behavior. Despite its relevance, only a few studies have adopted a person-centered approach to examine the functional heterogeneity of NSSI. Given a documented second peak of NSSI during emerging adulthood, latent profile analysis was used to examine the multifaceted nature of NSSI-related motivations among emerging adult university students. Of the 1378 university student respondents, analyses were restricted to the 161 individuals (11.7%) who reported engaging in NSSI within the past month and fell within the emerging adulthood age range (18–25 years). In the examined group, 81.4% were female and 79.5% were enrolled in a bachelor’s degree program (M_*age*_ = 21.18 years, *SD* = 1.64). Based on endorsement patterns for 13 NSSI functions, two comprehensible classes emerged. The Multi NSSI functions class (15.6%) exhibited medium to high scores across both intrapersonal and interpersonal NSSI functions. In contrast to the other distinct profile (Only emotion regulation class; 84.4%), the more functionally diverse group demonstrated greater vulnerability to negative emotional states and certain risky behaviors. The findings highlight this distinct subgroup, which merits special attention, as their engagement in NSSI for multiple motivations indicates a particularly elevated risk of poor mental health.

## Introduction

Given the elevated prevalence of nonsuicidal self-injury (NSSI) and its co-occurrence with other harmful behaviors within the university student population (La Guardia et al., 2020), there is a pressing need for in-depth examination of these maladaptive coping strategies. Despite this, the motivational underpinnings of NSSI remain an understudied aspect in this context, even though identifying distinct motivational patterns could offer critical insight into the psychological mechanisms underlying such harmful behaviors. In response to this gap, the present study applies a person-centered approach to identify distinct subgroups among emerging adult university students currently engaging in NSSI and subsequently compares these subgroups in terms of co-occurring negative emotional states, risky behaviors and indicators of NSSI severity.

NSSI covers a range of intentional behaviors (e.g., hitting, cutting, scratching, severe pinching, burning) that cause immediate direct physical harm to oneself but are not based on suicidal intent (Nock, 2010). NSSI is present not only in clinical but also in nonclinical populations and is identified as a major health problem in all settings (Liu, 2023). On the other hand, research is consistent in that NSSI acts occur most frequently in adolescence (Brown & Plener, 2017) and young adulthood (Wester et al., 2018). Systematic literature reviews from the past decade and a half have highlighted that the lifetime prevalence of NSSI among adolescents is high in both Europe and North America (Brunner et al., 2014), as well as in developing countries (e.g., Mexico, India, China; Mannekote Thippaiah et al., 2021), reaching 20%, and in some cases even 40–50%. During emerging adulthood—particularly among university students, who represent a unique subgroup of emerging adults—the prevalence rates of NSSI remain similarly high. A systematic review indicates a prevalence of nearly 40% among students in higher education (Cipriano et al., 2017). In the university population, lifetime history of NSSI is estimated at around 20% at the time of college entry (Kiekens et al., 2023). Outside of Europe and North America, lifetime prevalence rates of NSSI among university student populations are typically reported to be around 20% (Qu et al., 2023). However, findings regarding the 12-month incidence of NSSI are heterogeneous. Some studies indicate a lower prevalence, typically around 10% (Kiekens et al., 2019), whereas other studies report substantially higher rates. In a recent Spanish university sample, 30% of students reported engaging in NSSI five or more times within the past year (Schmidt et al., 2024). A large sample randomized trial found that ¾ of self-injurious US college students repeatedly engaged in NSSI, with nearly one in three admitting to engaging in NSSI for the first time in late adolescence or early young adulthood (Whitlock et al., 2006). Moreover, the literature indicates a clear trend of a radical increase in NSSI prevalence among young adults over the past decade and a half, with the lifetime prevalence among first-year US college students increasing from 16% to 45% between 2008 and 2015 (Wester et al., 2018). Nonetheless, it is important to emphasize that a recent one-year longitudinal study (Farrell et al., 2024) demonstrated a decline in NSSI engagement over the course of the first academic year, as revealed through latent growth curve modeling. In this study, the point prevalence of NSSI was notably high, reaching 35% among first-year students.

During the university years, empirical findings indicate not only an elevated prevalence of NSSI but also increase engagement in a range of risky behaviors, including substance use and abuse, tobacco use, unprotected sexual activity, reckless driving (Rivers et al., 2013), and excessive social media consumption (Salari et al., 2025). This life stage is also characterized by an increased prevalence of mental illness symptoms and elevated levels of stress (Auerbach et al., 2018). Moreover, longitudinal studies suggest that these associations are bidirectional (Boyne & Hamza, 2022), increased psychological distress raises the risk of NSSI, while engaging in NSSI further exacerbates emotional distress. In this context, it is important to note that among university students, NSSI has been found to be associated with a history of mental disorders (da Silva Bandeira et al., 2022); current difficulties in emotion regulation and interpersonal relationships (Farrell et al., 2024); symptoms of mental illness—particularly depression (Park, 2025); a significantly increased risk of suicidal self-injury (da Silva Bandeira et al., 2022); and various forms of risky behavior (Serras et al., 2010). The heightened vulnerability of the young adult university population to such behaviors and mental health symptoms can be attributed to the uncertainties inherent in transitional life stages. This transitional nature is, on the one hand, developmental: university attendance typically coincides with the life phase referred to by Arnett (2000) as emerging adulthood, encompassing the age range of 18 to 25 years. This period constitutes an in-between phase between the conclusion of adolescence and the assumption of adult roles and responsibilities. The developmental ambiguity of this stage is further reinforced by the internalization of the student identity, which may both buffer and intensify psychosocial challenges. During emerging adulthood, the university environment provides a central context for identity development. Identity formation actively occurs across multiple domains, including career planning and commitment, professional and personal interests, worldview, religious and political beliefs, becoming independent, peer networks, and romantic relationships. In each of these domains, individuals may experience stagnation, prolonged exploration, or identity diffusion, which can serve as an increased source of intrapersonal stress (Arnett, 2015). On the other hand, the university period also represents a transition in terms of psychosocial (e.g., financial and relational) stressors and life demands. In many cases, it involves separation from supportive familial and peer networks, while simultaneously introducing the individual into a performance-oriented context characterized by heightened academic and social pressures. This confluence of factors frequently contributes to increased levels of psychological distress and maladaptive coping strategies (Pedrelli et al., 2015). To better understand NSSI behavior, it is particularly important to examine its hypothesized antecedents and the motivations underlying it, that is, the functions that NSSI serves. In general, NSSI can be understood as a behavioral manifestation of a maladaptive coping style, although the underlying causes of such behavior are highly diverse (Klonsky, 2007). Several theoretical frameworks have attempted to categorize and explain these causes. The Four Function Model (FFM; Nock & Prinstein, 2004) identifies four primary motivational functions of NSSI, which can be conceptualized along two axes: the first distinguishes whether the behavior is driven by intrapersonal (also referred to as affective or automatic) or interpersonal (social) factors, and the second differentiates whether NSSI behavior serves a positively or negatively reinforcing function for the individual. Empirical research consistently highlights intrapersonal functions as the most common underlying motivation for NSSI (Brackman & Andover, 2017), with negative intrapersonal reinforcement—the reduction of aversive emotional states—being particularly prevalent. According to the dual model of NSSI functions (Klonsky & Glenn, 2009) two main factors underlie self-injury: intrapersonal and interpersonal motivations, while the positive and negative reinforcement dimension was not found to be relevant. The intrapersonal factor, which reflects motivations for regulating emotions, and the interpersonal factor, which reflects motivations related to coping with peer or other social experiences, encompass additional functional aspects of self-harm. The intrapersonal superordinate factor comprises five specific functions: (1) affect regulation, aimed at reducing negative emotional states (e.g., anger, sadness); (2) self-punishment, serving to direct anger or disgust toward oneself; (3) marking distress, used to communicate psychological pain through NSSI; (4) anti-dissociation, which involves generating feelings through NSSI to counter experiences of emptiness, depersonalization, or derealization; and (5) anti-suicide, which can help reduce intrusive suicidal ideation and/or the urge to attempt suicide. According to a meta-analysis summarizing several relevant studies, intrapersonal functions of NSSI occur more frequently than interpersonal functions, with the affect regulation function identified as the most prevalent intrapersonal NSSI motivation (Taylor et al., 2018). In a partly similar way, other reviews (e.g., Klonsky, 2007) found the two most common functions underlying NSSI to be affect regulation and self-punishment. Moreover, these two NSSI motivations are closely linked, often appearing together in the background of self-injury, as NSSI acts can reduce self-punitive negative emotions evoked by strong self-critical thoughts (Chester et al., 2015). Intrapersonal functions of NSSI were found to be positive predictors of NSSI severity (Brausch & Muehlenkamp, 2018). The much less frequent interpersonal functions integrate eight factors. Self-injury may provide an opportunity (1) to regulate interpersonal boundaries; (2) to replace care provided by others (self-care); (3) to seek extreme sensory experiences (sensation seeking); (4) to connect with others (peer bonding); (5) to communicate negative feelings and attract attention from others (interpersonal influence); (6) to express toughness; (7) to articulate revenge towards the environment; and 8) to demonstrate autonomy to the others. After summarizing several studies, interpersonal influence was found to be the most common interpersonal function, whereas communicating autonomy, expressing peer attachment, and sensation seeking through NSSI appeared as rarely occurring functions in the research (Taylor et al., 2018). Empirical evidence has also shown that each of these thirteen NSSI functions can be present simultaneously during a self-injurious episode (Klonsky, 2011).

Although the two-factor structure has been confirmed by all subsequent studies, the picture of which functions are subordinated to the two robust main factors (intra- and interpersonal) is more diverse. For example, some research identified self-care as part of the intrapersonal factor (e.g., Pérez et al., 2020), others as a component of the interpersonal factor (e.g., Bildik et al., 2013), while others have described it as a function that loads on both factors (e.g., Klonksy et al., 2015). This example highlights that the theoretical concept can become nuanced in empirical testing. One possibility is to explore how different functions group together in order to identify distinct subgroups of people engaging in NSSI. A useful tool for this could be latent class or profile analysis (LCA or LPA, respectively), which is a person-centered approach that seeks to determine whether individuals can be classified into distinct, homogeneous subgroups based on certain characteristics (called indicator variables). Ultimately, this method helps us to understand the heterogeneity of NSSI by identifying subgroups (classes) that exhibit similar patterns of the indicator variables. Furthermore, the method also enables the identification of differences between the latent classes using other relevant variables, thus validating the classes.

It is important to note that, in the context of NSSI, only few studies have employed person-oriented approaches (e.g., LCA or LPA), particularly in comparison to the predominance of variable-centered approaches within the college student population. A brief overview of person-oriented analyses in the field of NSSI reveals that several studies have identified four distinct subgroups within young adult samples, primarily differentiated by severity (Peterson et al., 2019). One consistently identified subgroup is characterized by engaging in multiple NSSI methods and primarily engages in NSSI for intrapersonal reasons. This so-called “multimethod” group tends to exhibit the poorest mental health outcomes (Bracken-Minor et al., 2012). The more severe NSSI group typically comprised around 10% of the studied samples. At the other end of the spectrum lies a subgroup characterized by low levels of NSSI episodes and methods, and subtle NSSI-related motivation, with relatively few psychological symptoms (Singhal et al., 2021). This least severe NSSI group tends to have the largest sample size across studies, generally ranging between 20% and 70%. Between these two extremes, there are typically subgroups characterized by moderate NSSI episode frequency and versatility (i.e., number of NSSI methods used; Case et al., 2020).

Based on the current literature, only a few studies have examined the heterogeneity of NSSI functions among college students using a person-centered approach. In such an analysis (Case et al., 2020), in addition to NSSI functions, several other NSSI-related variables (e.g., lifetime frequency, past-year frequency, number of methods, and perceived pain during NSSI) were also assessed to derive subgroups. Alongside groups characterized by mild, moderate, and severe severity levels, a distinct group was identified that engaged in NSSI for multiple reasons at a moderate level (“moderate multiple functions” NSSI group). In the “mild” group, all NSSI characteristics, including functions, were present to the lowest extent. These indicators were more elevated in the “moderate” group, while the “moderate multiple functions” group differed from the “moderate” group by exhibiting a broader range of NSSI motives, particularly interpersonal functions. The “severe” NSSI class demonstrated the most severe suicide risk and NSSI indicators, including a prominently intrapersonal functional background. These findings are highly consistent with an earlier study conducted among young adults. In the “multiple functions/anxious” group, NSSI was characterized by high versatility as well as the presence of both intra- and interpersonal functions. In contrast, the “automatic functions/suicidal” group primarily engaged in cutting due to intrapersonal reasons; this latter group exhibited the highest levels of psychopathology (Klonsky & Olino, 2008).

A study conducted on a sample of university students with a lifetime history of NSSI used K-means cluster analysis based on emotion regulation, alexithymia, and coping strategies to identify “emotion profiles”. Three profiles emerged: individuals with no emotion difficulties, those with moderate difficulties in emotion regulation, and those with generalized difficulties in regulating emotions. According to the results, individuals with generalized emotion regulation difficulties were more likely to have engaged in five or more self-injury episodes during the past year. For this group, the affect regulation and self-punishment functions of NSSI were particularly important in driving self-injurious behavior, and compared to the other two groups, they engaged in significantly more risky behaviors (alcohol use and eating problems) and exhibited higher levels of psychological distress as well as borderline symptomatology (Christoforou et al., 2021). Similarly, among university students who reported NSSI during the past year, three groups emerged with respect to emotion regulation: low, average, and high difficulties. The NSSI group with high emotion regulation difficulties (referred to as the “dysregulated” class) exhibited more interpersonal difficulties (i.e., relationship problems between parents and children) and engaged in a greater variety of NSSI methods. In this group, the sensation-seeking function of NSSI was higher compared to the low- and average-difficulties groups (Guérin-Marion et al., 2021). In a two-year longitudinal design as well, three stable profiles emerged with respect to the severity of NSSI and associated psychopathology (high-risk, middle, and low profile). Members of the “high-risk” profile were more likely to engage in severe forms of NSSI (e.g., cutting), were characterized by elevated emotional difficulties, and reported lower perceived social support, particularly from friends. Notably, in this group, interpersonal motivations played a particularly prominent role in NSSI compared to intrapersonal motivations. Among the three profiles, the “high-risk” profile exhibited the highest levels of depression, anxiety, stress, and borderline psychopathological symptoms, and its members were also more likely to report a past suicide attempt than individuals in the other two groups (Schmidt et al., 2024).

However, no person-centered studies to date have exclusively identified distinct heterogeneous subgroups based solely on NSSI functionality. It has been well established that individuals may engage in NSSI for multiple reasons simultaneously (Klonsky, 2007). A systematic review synthesized findings from 21 studies that had identified NSSI subtypes, aiming to integrate the inconsistent number and characteristics of these subtypes into a coherent theoretical framework. None of the reviewed studies specifically investigated groups derived exclusively from NSSI functions using a person-centered approach; instead, each study identified NSSI function groups together with multiple, and often differing, additional variables. This type of heterogeneity posed a significant challenge for synthesizing the results. As an outcome, the literature review concluded that two primary dimensions emerged as most salient in distinguishing NSSI subtypes: the endorsement of various NSSI functions and psychological pain (Wang et al., 2024). Across the included studies, greater endorsement of NSSI functions was consistently associated with increased NSSI severity and more adverse psychopathological outcomes. According to the Benefits and Barriers Model, the number of NSSI functions underlying an individual’s self-injurious behavior is associated with the perceived “benefits” expected from engaging in such behavior (Hooley & Franklin, 2018). Accordingly, individuals who engage in NSSI with a wide range of motivations tend to exhibit poorer psychological functioning, whereas those with few or limited motivations can be described as displaying an experimental pattern of NSSI, typically accompanied by fewer emotional and interpersonal difficulties. All these findings clearly suggest that it is worthwhile to identify latent groups based solely on the set of NSSI motivations, in order to interpret the different patterns of their co-occurrence (Wang et al., 2024).

## Current Study

To date, only a few studies have examined distinct patterns of NSSI functions. However, investigating the motivational basis underlying NSSI is essential to unravel the possible roots of self-harm. This is particularly important during youth, when NSSI is highly prevalent and often escalating in nonclinical samples. Furthermore, experimentation in risky behaviors is highly common during emerging adulthood. In line with this, this study aims to identify latent classes in a sample of emerging adult university students who have actively engaged in NSSI during the past month, based on the range of intra- and interpersonal functions they endorse. A person-centered approach, latent profile analysis (LPA), was used to explore the heterogeneity of the motivational background of NSSI that may characterize certain groups. In doing so, this study also seeks to reflect on the Benefits and Barriers Model and to empirically test its assumptions. These findings may enable the identification of subgroups of young adults at elevated risk of self-harm. In addition, potential covariates of latent profiles (i.e., age, gender, risky behaviors, mental illness symptoms, and severity indicators of NSSI) were applied in the comparison of groups emerging from NSSI functions. Based on earlier findings, it was hypothesized that emerging NSSI motivational profiles would vary according to intrapersonal and interpersonal functions and the intensity of these functions. It was also hypothesized that the high intrapersonal functions NSSI group would have poorer mental health compared with other classes.

## Methods

### Participants and Procedure

In a cross-sectional survey with a focus on mental health, risky behaviors and NSSI, between April 2023 and May 2024, Hungarian native-speaking students from different universities and diverse faculties in Hungary completed an online questionnaire package running on the Qualtrics platform. The recruitment was shared with students through various online university groups and student governments. Participation was voluntary and anonymous. There was no financial compensation for participation. The study was approved by the Institutional Review Board of ELTE Eötvös Loránd University Faculty of Education and Psychology, and the work was conducted in accordance with the Declaration of Helsinki (World Medical Association, 2013).

In total, 1778 completions were started during the year of data collection. Two students disagreed with the research criteria and were immediately directed to the end of the questionnaire, while 397 participants did not complete it . In total, 1379 people completed the entire questionnaire package. Of these, 1 person was excluded because of inconsistent completion of the questionnaire (e.g., indicating several episodes of self-harm and then categorizing themselves as not engaging in NSSI and writing obscene terms in the free text spaces). The final university sample thus consisted of 1378 students (*M*_age_ = 22.00 years, *SD* = 3.48; 74.2% females). Lifetime prevalence of at least one episode of NSSI was 40.5% (n = 558).

Analyses in the current study were limited to university students who reported at least one NSSI episode in the previous month (engaging in current NSSI; e.g., Jarvi et al., 2017) and were in the emerging adulthood life stage, i.e., between 18-25 years old (Arnett, 2000). We restricted our sample to individuals who had engaged in NSSI within the past month, as most of our assessed variables similarly targeted recent risky behaviors and psychological distress symptoms. This focus allows for a more accurate examination of the relationship between current NSSI behavior and present psychological functioning. Additionally, it helps minimize the influence of developmental or maturational factors, which are difficult to control for in a cross-sectional design without longitudinal data. Twelve percent (n = 166) of the total sample could be classified in this group. However, 4 students did not complete the motivation part of the Inventory of Statements About Self-Injury (ISAS; Klonsky & Glenn, 2009) questionnaire and 1 student clearly indicated that she had equated NSSI with self-starvation. As self-starvation, unlike NSSI, does not result in physical impairment immediately but in the longer term, it cannot be considered as NSSI in the strict sense (St. Germain & Hooley, 2012). Self-starvation is commonly classified under the umbrella term of indirect self-injury (ISI; Møhl, 2020). On this basis, these 5 students were excluded from the analysis.

Therefore, the current NSSI sample, on which the analyses were carried out, eventually included 161 students (11.7% of the total sample). The mean age of this group was 21.18 years (*SD* = 1.64), with an age range between 18 and 25. Most were female (81.4%; n = 131), 18.8% were male (n = 27), while 3 respondents (1,9%) classified themselves in other gender category (1 trans men, 1 nonbinary individual, and 1 demigirl). Nearly two-thirds of the sample (62.7%; n = 101) reported living in the capital, 13% (n = 21) in other large cities, approximately 15% (14.9%; n = 24) in small towns, and 9.3% (n = 15) in villages. Additionally, two-thirds of the sample (63.4%; n = 102) reported working while attending university, whereas 36.6% (n = 59) focused solely on their studies. Nearly half of the participants (49.1%; n = 79) were single, while 18% (n = 29) reported cohabiting with a partner, and 32.9% (n = 53) had a partner but did not live together. The vast majority of these students were enrolled in bachelor’s degree programs (79.5%; n = 128), while 9.9% (n = 16) were studying at the master’s level, another 9.9% (n = 16) were enrolled in undivided programs, and 0.6% (n = 1) were pursuing doctoral studies.

### Measures

#### Nonsuicidal self-injury

The Inventory of Statements About Self-Injury (ISAS; Klonsky & Glenn, 2009) was used to scan several aspects of NSSI. As an initial step, following the clear definition of NSSI, the questionnaire measures the lifetime frequency of episodes of 12 different NSSI methods (i.e., cutting, biting, burning, carving, pinching, pulling hair, severe scratching, banging or hitting self, interfering with wound healing, rubbing skin against rough surface, sticking self with needles, and swallowing dangerous substances). Subsequently, additional NSSI methods not previously indicated can also be reported, along with their frequency of occurrence. After the approximate number of episodes estimated by the respondents, those who reported at least one episode engaging in any NSSI method continued to complete the full questionnaire. This is followed by some questions that assess different additional factors of NSSI acts such as whether the person experienced pain or was alone during self-harm, whether the person wanted to stop self-harm or told someone that they engaged in self-harm. These questions were not used in our analysis. The ISAS also asks how old the person was when they first and last engaged in NSSI.

Finally, the last major section of the questionnaire measures the functions that may be motivating NSSI. The brief 26-item version of the motivational section of the ISAS (Washburn et al., 2012) was utilized. Thirteen functions can be identified, which fall into two broader intrapersonal and interpersonal categories (Klonsky & Glenn, 2009). The intrapersonal functionality factor condenses self-regulation (i.e., affect-regulation, anti-dissociation, anti-suicide, marking distress, and self-punishment) aspects of NSSI. While interpersonal functionality factor covers the social perspectives of NSSI motivations (i.e., autonomy, interpersonal boundaries, interpersonal influence, peer-bonding, revenge, self-care, sensation seeking, and toughness). Internal consistency was acceptable both for intrapersonal (α = 0.80), and interpersonal factors (α = 0.88) in the original long form of the ISAS, based on a young adult college sample (Klonsky & Glenn, 2009). Similar results were observed in the present study (α_intrapersonal_ = 0.75, and α_interpersonal_ = 0.84). Each item is rated on a 3-point scale (0 = not relevant, 1 = somewhat relevant, 2 = very relevant). In our research, several severity indicators of NSSI were adopted, i.e. (1) the number of NSSI methods engaged in; (2) the lifetime number of NSSI episodes; and (3) whether someone engaged in NSSI occasionally (1–9 self-injurious episodes) or repetitively (10 or more episodes; Gratz et al., 2015). Participants were categorized based on whether they had engaged in NSSI within the past month (current NSSI) or more than a month ago (former NSSI).

#### Mental iIllness symptoms

The 21-item version (Henry & Crawford, 2005) of the Depression Anxiety and Stress Scales (DASS; Lovibond & Lovibond, 1995) was used to assess basic psychological distress symptom domains. On a four-point scale (0 = never, 1 = sometimes, 2 = often, 3 = almost always), symptom presence of depression (e.g., dysphoria, hopelessness), anxiety (e.g., autonomic arousal, situational anxiety), and stress (e.g., irritability, difficulties of relaxing) were evaluated over the past week. A minimum score of 0 and a maximum score of 21 can be obtained on each scale. By summing the three subscales, a total symptom score can also be calculated. The questionnaire has no diagnostic value on its own, however shows the extent of the psychological distress present. The reliability of the instrument is excellent (α = 0.93 for the total scale), as is its validity (Henry & Crawford, 2005). Similarly, reliability for the total scale was excellent among undergraduates (ω = 0.96; Kia-Keating et al., 2018), as well as in the present study (α = 0.92).

#### Sleep problems

The self-reported Athens Insomnia Scale, based on ICD-10 criteria, is a valid and reliable measure (α = 0.89) of various sleep problems and the severity of insomnia (Soldatos et al., 2000). Sleep quality is structured around 8 sets of questions (sleep induction, awakening at night, early awakening, short sleep duration, poor sleep quality, and daytime effect of sleep disturbances, like wellbeing, functioning, and sleepiness) on a four-point scale ranging from no problems (0) to severe problems (3). The total score of the whole questionnaire reflects the severity of insomnia (Soldatos et al., 2000). The reliability of the total scale was good in the recent study (α = 0.80), consistent with previous findings among university students (e.g., α = 0.84 in Busa et al., 2023).

#### Risky behaviors

Four domains of risky behavior were included in the assessment.

##### Alcohol use

The Alcohol Use Disorder Identification Test (AUDIT; Saunders et al., 1993) is a worldwide used alcohol screening instrument. The self-administered measurement asks 10 questions about alcohol-related problems (alcohol dependence and consequences of harmful and hazardous alcohol consumption) over the past year. Items were assessed on a 5-point scale (0–4), with response options differing by item content. The total score ranges from 0 to 40. The reliability of the AUDIT was excellent in the original study (α = 0.93; Saunders et al., 1993) and good in higher education samples (α = 0.81; Skogen et al., 2024), as well as in the recent study (α = 0.83).

##### Smoking

As no validated smoking assessment questionnaires were available in Hungarian, the regularity of smoking was assessed with a single question (“Do you smoke regularly or occasionally (cigarettes, cigars, tobacco, e-cigarettes)?”) formulated by the authors, offering three response options (yes, regularly (every day); yes, occasionally; no). Participants who reported smoking were additionally asked to indicate the average number of cigarettes they smoked per day.

##### Problematic internet use

The 9-item version of the Problematic Internet Use Questionnaire (PIUQ; Demetrovics et al., 2008) was used to map the problems associated with excessive and problematic use of the Internet. The self-reported items can be rated on a 5-point scale (1 = never, 2 = rarely, 3 =sometimes, 4 = often, 5 = always/almost always) of how typical the statements are of the person in general. In addition to the total score, three subscales (obsession, neglect of daily tasks, control problems) can be distinguished. The reliability of the total PIUQ was good in the original study (α = 0.87; Demetrovics et al., 2008), in an American college student sample (α = 0.91; Kelley & Gruber, 2010), and in our research (α = 0.82).

##### Social media addiction

Finally, the self-reported, six-item Bergen Social Media Addiction Scale (BSMAS; Andreassen et al., 2016) measured social media addiction symptoms over the past year. Items were rated on a 5-point scale (1 = very rarely, 2 = rarely, 3 = sometimes, 4 = often, 5 = very often), with higher scores indicating greater addiction severity. The BSMAS is a reliable measurement (Cronbach α was 0.88 in the original study (Andreassen et al., 2016), 0.86 among young adults (Shin, 2022), and 0.80 in the present study).

### Data Analysis

Data were analyzed using SPSS 30.0 (IBM SPSS, IBM Corp., Armonk, NY) and Mplus 8.10 software packages (Muthén & Muthén, 1998–2007).

To begin with, descriptive statistics, and the bivariate Pearson’s correlation coefficients between the study variables were calculated. Following that, latent profile analysis (LPA) was utilized to identify homogeneous subgroups (latent classes) of participants based on their scores on the 13 functions of NSSI. The optimal number of classes was determined by considering models with different numbers of latent classes using the Akaike Information Criterion (AIC), Bayesian Information Criterion (BIC), Sample Size Adjusted Bayesian Information Criterion (SSA-BIC), and Lo-Mendel-Rubin Adjusted Likelihood Ratio Test (LMRT). Lower values of AIC, BIC, and SSA-BIC are indicative of a superior fit, and a non-significant LMRT suggests that the inclusion of an additional latent class would not have improved the fit of the model. The entropy index, with higher values (closer to 1), indicates a more precise classification of participants; for instance, values around 0.8 are considered indicative of high entropy (Clark & Muthén, 2009). In the next step, differences between the latent classes were examined in terms of demographic variables, NSSI severity indices (e.g., number of methods and number of episodes), emotional distress, sleep problems, smoking, risky drinking, and problematic internet and social media use, employing the Bolck, Croon, and Hagenaars (BCH) method (Asparouhov & Muthén, 2014). Subsequently, multinominal logistic regression analysis employing the 3-step method was used to explore the relationship between the most likely latent class membership and covariates.

## Results

### Preliminary Analyses

#### Descriptive statistics of the study variables

Descriptive statistics and correlations between the study variables are presented in Table 1. Correlation coefficients were significant in the expected directions. Psychological distress symptoms (i.e., depression, anxiety, and stress) and risky behaviors (e.g., sleep problems, alcohol use, and problematic internet use) showed weak to moderate positive associations with each other.

**Table 1:** Descriptive Statistics and Correlations Between the Study Variables

Variable	M (SD)	1	2	3	4	5	6	7	8	9	10	11	12	13	14	15	16	17	18
1 Age	21.18 (1.64)	** –**																	
2 Gender		−0.01	** –**																
3 DASS Total	29.50 (13.60)	0.08	−0.10	** –**															
4 DASS Depression	9.45 (5.75)	0.07	0.04	** 0.86**	**–**														
5 DASS Anxiety	8.57 (5.38)	0.04	−0.09	** 0.86**	** 0.56**	** –**													
6 DASS Stress	11.48 (4.67)	0.10	−**0.24**	** 0.87**	** 0.62**	** 0.66**	**–**												
7 AIS Total	7.56 (4.19)	0.001	−0.03	** 0.59**	** 0.56**	** 0.50**	** 0.45**	** –**											
8 AUDIT Total	6.40 (5.50)	−0.01	0.15	** 0.21**	** 0.21**	** 0.21**	0.12	** 0.30**	**–**										
9 PIUQ Total	23.34 (6.46)	−0.01	0.01	** 0.27**	** 0.24**	** 0.21**	** 0.24**	** 0.25**	*** 0.20***	** –**									
10 PIUQ Obsession	5.86 (2.88)	−0.01	0.003	** 0.20**	*** 0.16***	0.14	** 0.22**	0.15	** 0.22**	** 0.79**	**–**								
11 PIUQ Neglect	8.45 (2.45)	−0.07	0.02	** 0.23**	** 0.25**	*** 0.16***	*** 0.18***	** 0.23**	** 0.21**	** 0.81**	** 0.45**	** –**							
12 PIUQ Control problems	9.03 (2.73)	−0.01	−0.01	** 0.22**	*** 0.18***	** 0.21**	*** 0.17***	** 0.23**	0.05	** 0.81**	** 0.40**	** 0.55**	**–**						
13 BSMAS Total	14.20 (4.99)	−0.04	−0.08	** 0.24**	** 0.24**	*** 0.19***	*** 0.19***	** 0.27**	** 0.26**	** 0.77**	** 0.66**	** 0.58**	** 0.59**	** –**					
14 NSSI Episodes	1763,05 (10434,24)	*** 0.18***	0.01	*** 0.19***	*** 0.18***	0.15	0.15	*** 0.17***	*** 0.16***	0.03	0.007	0.02	0.04	−0.03	**–**				
15 NSSI Repetitive		0.01	−0.01	−0.09	−0.02	−0.09	−0.14	0.05	−0.08	0.001	−0.02	−0.04	0.06	0.09	0.04	** –**			
16 NSSI Methods	4.54 (2.87)	0.06	−0.02	** 0.31**	** 0.30**	** 0.27**	** 0.20**	** 0.28**	** 0.23**	** 0.21**	*** 0.17***	*** 0.19***	0.14	** 0.27**	*** 0.16***	** 0.24**	** –**		
17 NSSI Intrapersonal functions	9.27 (4.35)	0.02	−0.09	** 0.51**	** 0.46**	** 0.45**	** 0.40**	** 0.38**	** 0.26**	** 0.26**	** 0.24**	** 0.21**	*** 0.18***	** 0.31**	*** 0.16***	−0.07	** 0.36**	** –**	
18 NSSI Interpersonal functions	4.55 (4.96)	0.001	0.09	** 0.43**	** 0.31**	** 0.41**	** 0.39**	** 0.30**	** 0.34**	** 0.37**	** 0.39**	** 0.25**	** 0.25**	** 0.38**	0.02	−***0.18***	** 0.29**	** 0.45**	** –**

N = 161. Gender was coded 1 = women and 2 = men. Occasional NSSI = 1 and Repetitive NSSI = 2. Pearson correlations were calculated for continuous scores, point-biserial correlations were calculated for correlations involving dichotomous variables (gender and NSSI Repetitive vs. Occasional)

Correlations that are significant at the 0.05 level are shown in ***bold italics***. Correlations that are significant at the 0.01 level are shown in **bold** (two-tailed)

*DASS* depression anxiety and stress scales 21-item version; *AIS* athen insomnia scale; *AUDIT* alcohol use disorders identification test; *PIUQ* problematic internet use questionnaire; *BSMAS* bergen social media addiction scale; *NSSI* nonsuicidal self-injury

Regarding gender and age, women reported higher levels of stress, while older emerging adult university students reported a greater number of NSSI episodes. In terms of NSSI severity indicators, the following findings were highlighted: (1) The number of NSSI methods showed weak to moderate correlations with the examined psychopathological variables, including psychological distress symptoms and risky behaviors. (2) The frequency of NSSI episodes was weakly associated with depressive symptoms, sleep difficulties, and problematic alcohol use. (3). Finally, whether individuals engaged in occasional or repetitive NSSI was not associated with any of the assessed psychopathological indicators.

Both the intrapersonal and interpersonal NSSI function factors were moderately associated with psychological distress symptoms, sleep problems, problematic drinking, and internet addiction. Intrapersonal motivations were weakly associated with a higher number of NSSI episodes and moderately associated with greater NSSI versatility. Interpersonal functionality was also moderately related to NSSI versatility and showed a weak association with occasional self-injury. The two functional domains were moderately and positively correlated with each other.

#### Descriptive statistics of current NSSI behaviors

Most participants in the current NSSI sample (67.7%; n = 109) engaged in NSSI from 1 to 5 days in the previous month, another 13.7% (n = 22) reported NSSI between 6 and 10 days, 5% (n = 8) between 11 and 15 days, while further 13.7% (n = 22) more than 15 days. Undergraduates reported a mean age of 11.55 years (SD = 4.12) at their first engagement in NSSI, with the highest prevalence observed between ages 10 and 16. The most common NSSI methods, besides interfering with wound healing (66.5%; n = 107), were biting (56.5%; n = 91), pinching (56.5%; n = 91), banging or hitting self (53.4%; n = 86) and severe scratching (50.3%; n = 81). Cutting was reported in 34.8% of the cases (n = 56). Swallowing dangerous substances was the only method with a prevalence below 10% (5.6%; n = 9). On average, the sample applied 4 different NSSI methods (*M* = 4.54; *SD* = 2.87; ranged between 1–13 methods). The average lifetime number of NSSI episodes ranged from 1 to 121,605 (*M* = 1763,05; *SD* = 10434,24). The overwhelming majority (94.4%; n = 152) engaged in repetitive NSSI ( ≥ 10 lifetime episodes; Gratz et al., 2015). The most prominent NSSI functions were emotion regulation (*M* = 2.94; *SD* = 1.32), self-punishment (*M* = 1.95; *SD* = 1.56), managing suicidal thoughts (*M* = 1.72; *SD* = 1.00), and generating emotions, such as alleviating dissociative states (*M* = 1.53; *SD* = 1.49). In contrast, the least endorsed functions were revenge (*M* = 0.34; SD = 0.89) and peer bonding (*M* = 0.37; *SD* = 0.84).

### Latent Profile Analysis

**Fig. 1:**
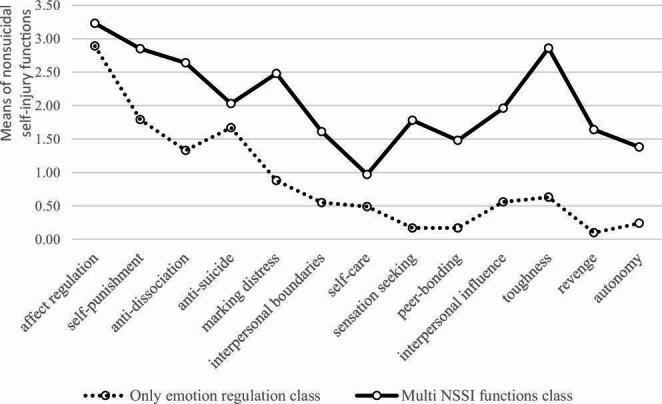
The two latent classes of NSSI functions

Based on the fit indices of the LPA, the 2-class solution proved to be the best model (Table 2). In case of the three-class model, the LMRT index was no longer significant (*p*  =  0.257), suggesting the two-cluster over the three-class solution. Table 3 displays the descriptive statistics of NSSI functions by latent classes. To facilitate interpretation, the means of each NSSI function for the 2-class solution are displayed in Fig. 1. Table 4. reports the descriptive statistics for the 2 classes: a group whose members identified emotion regulation as the primary function of NSSI (85.1%; n = 137); a group whose members had highly polymorphic motivations (14.9%; n = 24).

**Table 2:** Fit indices for the latent profile analysis of nonsuicidal self-injury functions

	AIC	BIC	SSA-BIC	Entropy	LMRT	p
**2 class**	**5859.5**	**5982.7**	**5856.1**	**0.983**	**400.243**	**0.025**
3 class	5658.6	5825.0	5654.0	0.976	225.741	0.257

The selected model is boldfaced

*AIC* akaike information criteria; *BIC* bayesian information criteria; *SSA-BIC* sample size adjusted bayesian information criteria; *LMRT* Lo-Mendel-Rubin adjusted likelihood ratio test

**Table 3:** The means (with standard deviation) of nonsuicidal self-injury functions by latent classes

	Only emotion regulation class	Multi NSSI functions class
affect regulation	2.89 (1.30)	3.23 (1.30)
self-punishment	1.79 (1.50)	2.85 (1.50)
anti-dissociation	1.33 (1.41)	2.64 (1.41)
anti-suicide	1.67 (0.98)	2.03 (0.98)
marking distress	0.88 (1.09)	2.48 (1.09)
interpersonal boundaries	0.55 (0.84)	1.61 (0.84)
self-care	0.49 (0.95)	0.97 (0.95)
sensation seeking	0.17 (0.52)	1.78 (0.52)
peer-bonding	0.17 (0.70)	1.48 (0.70)
interpersonal influence	0.56 (1.00)	1.96 (1.00)
toughness	0.63 (1.00)	2.86 (1.00)
revenge	0.10 (0.70)	1.64 (0.70)
autonomy	0.24 (0.67)	1.38 (0.67)

*NSSI* nonsuicidal self-injury

**Table 4:** Descriptive statistics and group comparison for nonsuicidal self-injury functions: a two-class solution

	Class1 (N = 137) – Only emotion regulation class	Class 2 (N = 24) –Multi NSSI functions class	Wald statistics (p)
Male (%)	19.7%	25.0%	0.30 (0.583)
Age, Mean (SE)	21.21 (0.14)	21.04 (0.29)	0.26 (0.610)
Regular smokers (%)	14.6%	20.8%	0.50 (0.480)
AIS Total score, Mean (SE)	7.14 (0.36)	9.92 (0.64)	14.28 (<0.001)
DASS Total score, Mean (SE)	27.61 (1.11)	40.11 (2.44)	21.69 (<0.001)
DASS Depression, Mean (SE)	8.92 (0.49)	12.47 (1.03)	9.71 (0.002)
DASS Anxiety, Mean (SE)	7.80 (0.44)	12.84 (1.01)	20.89 (<0.001)
DASS Stress, Mean (SE)	10.89 (0.38)	14.80 (0.87)	16.92 (<0.001)
AUDIT, Mean (SE)	6.43 (0.39)	9.59 (1.50)	4.12 (0.042)
BSMAS, Mean (SE)	13.65 (0.40)	17.26 (1.16)	8.56 (0.003)
PIUQ Total score, Mean (SE)	22.61 (0.51)	27.39 (1.53)	8.75 (0.003)
PIUQ Obsession, Mean (SE)	5.47 (0.22)	8.01 (0.75)	10.59 (0.001)
PIUQ Neglect, Mean (SE)	8.31 (0.20)	9.21 (0.57)	2.22 (0.136)
PIUQ Control problems, Mean (SE)	8.83 (0.23)	10.17 (0.52)	5.58 (0.018)
NSSI episodes, Mean (SE)	1819.18 (960.79)	1449.07 (604.482)	0.11 (0.745)
Repetitive vs. occasional (%)	95.6%	87.5%	1.36 (0.242)
NSSI methods, Mean (SE)	4.30 (0.23)	5.87 (0.78)	3.70 (0.054)

*SE* standard error; *AIS* athen insomnia scale; *DASS* depression anxiety and stress scales 21-item version; *AUDIT* alcohol use disorders identification test; *BSMAS* bergen social media addiction scale; *PIUQ* problematic internet use questionnaire; *NSSI* nonsuicidal self-injury

### Multinominal Logistic Regression to Predict Class Membership (N = 146)

In the next step, a multinomial logistic regression analysis was conducted (Table 5) , in which auxiliary variables, such as gender (male or female), age, NSSI methods, insomnia symptoms (AIS score), problematic internet use (PIUQ score), risky alcohol use (AUDIT score) and psychological distress (DASS-21 score) predicted class membership. Note, that in this analysis, only the significant variables from the univariate group comparison were included. A correlation analysis revealed a significant relationship between problematic internet use (PIUQ score) and problematic social media use (BSMAS score) (*r* = 0.765). To prevent multicollinearity, only PIUQ score was included in the prediction of class membership. Compared to the Only emotion regulation group (Class 1) only increased psychological distress was associated with being in group with Multi NSSI functions (Class 2).

**Table 5:** Multinomial logistic regression analysis to predict class membership of Multi NSSI functions group (N = 146)

Covariates	OR [95% CI]	p
Gender	1.05 [0.40–2.77]	0.914
Age	0.86 [0.63–1.17]	0.334
NSSI methods	1.01 [0.81–1.26]	0.908
AIS total score	1.02 [0.86–1.22]	0.786
**DASS total score**	1.08 [1.03–1.14]	**0.002**
AUDIT total score	1.06 [0.97–1.17]	0.218
PIUQ total score	1.08 [0.99–1.17]	0.071

Reference group is the Only emotion regulation class. Significant covariates are boldfaced

*AIS* athen insomnia scale; *DASS* depression anxiety and stress scales 21-item version; *AUDIT* alcohol use disorders identification test; *PIUQ* problematic internet use questionnaire; *NSSI* nonsuicidal self-injury

### Sensitivity Analysis

The analyses were repeated with grouping functions into intrapersonal and interpersonal functions. However, for the two-class solution, the LMRT index was not significant (p  =  0.500), suggesting that using the intra- and interpersonal function scales it is not possible to identify homogeneous subgroups (latent classes) of participants (Table 6).

**Table 6:** Fit indices for the latent profile analysis of intra- and interpersonal nonsuicidal self-injury functions

	AIC	BIC	SSA-BIC	Entropy	LMRT	p
2 class	1854.6	1876.1	1854.0	0.867	56.89	0.500

*AIC* akaike information criteria; *BIC* bayesian information criteria; *SSA-BIC* sample size adjusted bayesian information criteria; *LMRT* Lo-Mendel-Rubin adjusted likelihood ratio test

## Discussion

Despite a documented second, smaller peak of NSSI behaviors in young adulthood (Kiekens et al., 2019) and its high prevalence in university populations—with some studies even reporting an increasing trend (Wester et al., 2018)—relatively few studies have explored the heterogeneous nature of NSSI within emerging adult university samples. Conversely, given the clear evidence that most individuals who engage in NSSI do so for multiple reasons, it is recommended that the co-occurrence of specific functions also be examined, as these potentially distinct patterns may imply different prognoses and require different points of intervention (Klonsky et al., 2015). In addition, a review study suggests examining NSSI in terms of its functions, as NSSI performed for multiple motivations can be associated with more severe psychopathological outcomes (Wang et al., 2024). Considering all of these factors, using latent profile analysis, a person-centered methodological approach, the present study aimed to identify NSSI phenotypes based on intra- and interpersonal functions of NSSI among 18- to 25-year-old university students who reported engaging in NSSI within the past month. This temporal consideration is particularly relevant to class identification, as biopsychosocial changes, including neurological maturation, identity development, and shifts in social roles and relationship, can be especially rapid during emerging adulthood (Sawyer et al., 2018). Furthermore, the indicators of mental health and risky behaviors assessed in the present study were based on participants’ experiences in the past few weeks, which further underscores the importance of accounting for the timing-related sensitivity and developmental instability characteristic of this age group.

The lifetime prevalence of NSSI in the total sample of Hungarian emerging adult university students was 40.5%, while the point prevalence (NSSI within the past month) was 11.7%. Notably, the vast majority—approximately 95%—of those engaging in NSSI reported repetitive self-injury. The observed lifetime prevalence is entirely consistent with findings from similar studies conducted among university student populations (Wester et al., 2018). Furthermore, as in other university samples, the majority of students reported repetitive NSSI engagement. In contrast, considerably fewer studies have assessed the point prevalence of NSSI. Among first-year university students, a study reported a point prevalence of 25% (Hamza et al., 2021), whereas a more recent study found a prevalence of 35% (Farrell et al., 2024). Although the point prevalence observed in our study was lower, it is important to note that our research included students across all years of university, rather than being limited to first-year students. Previous research has documented a decreasing trend in NSSI across the first university year (Farrell et al., 2024), which has been attributed to students’ increasing adaptation to the challenges of university life (Conley et al., 2020).

The analyses focused on the subsample of individuals who had engaged in NSSI during the previous 30 days, representing 11.7% of the total sample. Within the subsample of individuals reporting recent NSSI, two distinct and interpretable classes emerged; however, these were not differentiated solely by the anticipated distinction between intra- and interpersonal motivations, but rather by the severity (i.e., characteristic presence) of the functions. One of the identified NSSI phenotypes, comprising the majority (85%) of university students who reported engaging in NSSI in the previous month, was characterized by intrapersonal motivations only—specifically affect regulation, self-punishment, anti-dissociation, anti-suicide, and marking distress. Interpersonal functions were minimally present in this group, suggesting that their self-injurious behavior was primarily driven by internal emotional regulation needs. Accordingly, this group was labeled the *Only emotion regulation group*.

In contrast, a smaller proportion of participants (15%) formed a distinct profile marked by high to moderate endorsement of all examined NSSI functions. This class, labeled the *Multi NSSI functions class*, appeared to engage NSSI for both intrapersonal and interpersonal reasons, indicating a broader functional repertoire that includes emotional regulation as well as social signaling and boundary-setting functions. Within this more functionally diverse group, the self-care function emerged as the least endorsed among the 13 assessed motivations. This finding aligns with previous variable-centered research (e.g., Taylor et al., 2018), which have consistently identified self-care as one of the least commonly endorsed NSSI functions. Our results support the notion that self-injury rarely serves as a substitute for care that individuals expect from their social environment. Even among individuals with a more complex NSSI profile, self-care does not appear to be a central motivational aspect. Comparing the two NSSI function subgroups, quantitative differences could not be found according to gender or age. These findings are consistent with previous latent class analyses conducted among adolescents, where age (Somer et al., 2015) and gender (Lloyd-Richardson et al., 2007) were not significantly associated with class membership and further support the notion that developmental and gender-related factors play a limited role in the heterogeneity of NSSI.

However, individuals belonging to the Multi NSSI functions group exhibit significantly poorer mental health. They report higher levels of depression, anxiety, and stress, experience poorer sleep quality, and engage in more risky behaviors, including increased problems with alcohol consumption, as well as obsessive use of the internet and social media. The Multi NSSI functions group did not show significant associations with severity indicators of NSSI such as number of lifetime episodes and related repetitive self-injury. However, at a trend-level toward statistical significance, it was observed that the Multi NSSI functions group employed a greater variety of NSSI methods compared to the Only emotion regulation group. Although, based on the existing literature, no study has specifically examined the phenotypes of NSSI functionality independently, a few analysis have incorporated NSSI functions as a key factor in subgroup formation. In our study, the identified Multi NSSI functions group—characterized as a more at-risk subgroup compared to the Only emotion regulation group—bears a strong resemblance to the Multiple functions/anxious group described in a study of young adults (Klonsky & Olino, 2008). That group engaged in a broader range of NSSI behaviors for both intrapersonal and interpersonal reasons, and reported significantly elevated levels of anxiety. It is also worth highlighting that this group represented 11% of the whole lifetime NSSI sample, a proportion that closely mirrors the Multi NSSI functions group’s representation in the current study. Furthermore, while both groups showed heightened psychological vulnerability, our findings extend beyond anxiety to include a broader spectrum of psychopathological indicators. Another potential parallel is the group identified among university students that exhibited a moderate level of NSSI engagement and severity, primarily driven by multiple, predominantly interpersonal functions (moderate multiple functions group; Case et al., 2020). The present findings are also consistent with person-centered approach studies (e.g., Schmidt et al., 2024) emphasizing the importance of interpersonal motivations among young people who engage in NSSI, particularly within more severe self-injuring groups. The results of the present study can provide empirical evidence for the proposition of the Benefits and Barriers Model, which suggests that individuals experiencing multiple emotional and social difficulties may alleviate the resulting stress through NSSI driven by multiple motivations, meaning that NSSI behavior holds greater perceived benefits for them (Hooley & Franklin, 2018).

The current findings support the conceptualization of emerging adulthood as an extended period of adolescence (Arnett, 2000), during which many young people engage in higher education. University life brings numerous novelties compared to previous life stages (e.g., increased autonomy, intensified performance demands, adaptation to new living environments), which are associated with elevated distress and negative emotions (Miller & Racine, 2022). The formation of student and adult identities, alongside detachment from parental control, imposes heightened demands for adaptation. Research links these challenges to elevated mental health problems within the university student population (Paiva et al., 2025). Moreover, coping with the increased distress associated with emerging adulthood tasks may facilitate the temporary use of maladaptive emotional and social regulatory behaviors, such as certain risky behaviors (e.g., psychoactive (Jackson et al., 2005) and behavioral addictions; Cheng et al., 2021; Liu et al., 2025), as well as NSSI (Kiekens et al., 2019). The transiently elevated prevalence of impulsive and risk-seeking behaviors can be understood within the framework of a neurobiological imbalance, whereby the limbic system—responsible for emotional and reward processing—develops more rapidly relative to the prefrontal cortex, which governs control processes (Steinberg, 2008). A meta-analysis synthesizing 25 studies, including samples of emerging adults, identified problem behaviors (e.g., substance misuse) as the strongest risk factors for NSSI (Wang et al., 2022), while another literature review found that depressive symptoms significantly increase the likelihood of NSSI onset (Hamza et al., 2012). It appears that these associations are more pronounced within the Multi NSSI class, suggesting that this group may be considered a more at-risk population in terms of NSSI.

In summary, two distinct latent classes were identified, primarily differing in the number and severity of NSSI functions. This finding suggests that the greater the number (i.e., diversity) of functions underlying an individual’s self-injurious behavior, and the stronger these functions are present, the more impaired their psychological functioning tends to be, and the more severe their NSSI may become. Therefore, the endorsement of multiple NSSI functions may serve as a meaningful indicator of NSSI severity. Alongside commonly recognized indicators—such as the total number of NSSI episodes, the distinction between experimental and repetitive engagement, and the number of NSSI methods used (i.e., versatility)—the presence of multiple concurrent NSSI functions (i.e., functional/motivational versatility) emerges as a salient and interpretable marker of NSSI severity. Previous variable-centered studies identified only intrapersonal motivations as significant positive predictors of NSSI severity (Brausch & Muehlenkamp, 2018). In contrast, the present person-centered approach extends these findings by highlighting the combined high presence of both intrapersonal and interpersonal motivations as a distinct NSSI severity indicator.

Using multivariate analysis, among the examined factors only psychological distress emerged as a significant predictor of membership in the Multi NSSI functions group. These findings highlight the importance of paying particular attention to university students exhibiting negative mood and anxiety symptoms, as well as experiencing elevated stress, in the context of NSSI prevention and intervention. Depression is often accompanied by emotional pain, feelings of helplessness, and anxiety, which affected individuals frequently attempt to regulate or alleviate through NSSI. However, in the long term, this coping strategy may contribute to the worsening of psychological problems. Based on the findings of the present study, although a cross-sectional study is unable to detect causality, it is important to highlight that elevated levels of negative affect and stress may contribute to the emergence of NSSI for multiple reasons—both intrapersonal and interpersonal in nature. This observation is in line with contemporary explanatory models of NSSI, which suggest that in the context of emotional overload and interpersonal difficulties, NSSI is maintained because it serves—at least in the short term—as an effective strategy for managing negative emotional states and coping with distressing social situations (Chapman et al., 2006; Nock, 2009). At the same time, the relationship between stress and NSSI is not unidirectional: distress may lead to NSSI as a means of release, but the act of NSSI itself can also generate stress for the individual. Thus, NSSI is not only a consequence but also a cause of stress, and this reciprocal relationship functions as a maintaining factor of self-injurious behavior. According to empirical evidence from the diathesis–stress model of NSSI, life stressors increase the risk of engaging in NSSI through various mediational pathways — such as psychological distress and emotion regulation difficulties (Voon et al., 2014), depressive cognitive style (Guerry & Prinstein, 2010), and reduced social support (Christoffersen et al., 2015). Based on the conclusions of a systematic literature review, distal stressors typically contribute to the onset and recurrence of NSSI through mediational pathways, whereas proximal stressors—most often interpersonal in nature—serve as precipitating factors for NSSI (Liu et al., 2016).

### Treatment Implications

In a longitudinal study conducted in a clinical setting, it was demonstrated that although NSSI functions tend to remain temporally stable within individuals, successful treatment led to a clear reduction in both intrapersonal and interpersonal functions. Notably, decreases in intrapersonal functions were particularly associated with marked improvements in clinical status (Victor et al., 2016). These considerations underscore the importance of assessing the motivational structure of NSSI in individual psychological care at the university level whenever self-injurious behavior emerges—alongside the already identified indicators of NSSI severity—and subsequently targeting these underlying factors in treatment. Particular vigilance is warranted when multiple intrapersonal and interpersonal motives are simultaneously present, as this may signal more severe NSSI and a poorer mental health status, especially in relation to co-occurring health-risk behaviors and negative emotional states. Treatment in this population may be particularly challenging due to the overdetermined nature of NSSI and the presence of comorbid psychopathologies (Klonsky & Olino, 2008), which are likely to have a mutually reinforcing effect. It is presumed that NSSI temporarily alleviates symptoms arising from associated psychological difficulties, while in the long term, it exacerbates them. Based on these considerations, reducing NSSI—particularly its motivational underpinnings—may be achieved through improving the negative emotional states present among those who engage in NSSI.

Based on the diathesis–stress model of NSSI, it is important to systematically assess proximal stressors stemming from the university environment and the challenges typical of emerging adulthood. Such stressors could be screened using negative life events questionnaires specifically designed for undergraduates or explicitly addressed during the counseling process, as distress triggered by these factors—particularly those of an interpersonal nature—may further increase the number of underlying factors contributing to NSSI. Accordingly, among undergraduates who engage in NSSI driven by multiple motivational factors, mapping their relational dynamics and, based on this, enhancing their peer relationships may provide substantial support in reducing both the interpersonal and intrapersonal motivational bases of NSSI. Community-building is already an important quality assurance objective for most universities; therefore, promoting the integration of students who experience difficulties in social adjustment—especially first-year students—into peer communities may also serve as an effective strategy for NSSI reduction. At the same time, it is important to emphasize that members of the Multi NSSI functions group likely warrant clinical psychological and psychiatric attention and, following identification as a high-risk group, should be directed toward appropriate care. In this regard, evidence-based methods that are structured and skill-based are particularly recommended. In a clinical psychology setting, a review study highlighted mentalization-based treatment (MBT) as an effective intervention for NSSI (Calati & Courtet, 2016), while depressive (Carey et al., 2025) and anxiety symptoms (Dai et al., 2025) can be effectively reduced through cognitive behavioural therapy (CBT) protocols. Dialectical behavior therapy (DBT; Linehan et al., 2015) may be particularly effective in addressing both intra- and interpersonal motivations for NSSI, as it targets difficulties in emotion regulation as well as social adjustment. Empirical evidence in university settings indicates that DBT is especially beneficial for students with pronounced psychopathological symptoms, including NSSI (Uliaszek et al., 2016). Participation in DBT groups fosters the development of close, supportive relational patterns, strengthening the basic psychological need for relatedness. The strengthening of relatedness, by reducing overall stress levels, serves as a protective factor against NSSI and its recurrence.

### Limitations

Although this study offers valuable insights into NSSI during emerging adulthood and highlights multiple underlying motivations as indicators of severity, several limitations should be acknowledged. Reliance on self-report data can lead to a distortion of recall or social desirability, especially on sensitive issues such as self-harm and risky behavior. In addition, the cross-sectional nature of the study limits the establishment of causal links. Longitudinal studies and comparative studies across different national samples are also required to demonstrate the validity and the relevance of the recently identified NSSI severity factor.

Third, although a relatively large sample of Hungarian university students from several different universities and majors was obtained, the number of the analyzed currently NSSI subsample was lower by nature, which should be an important consideration when drawing conclusions. This is particularly relevant from the perspective of the applied statistical method. For LPA, the overall sample size (N = 161) can be considered small (Nylund et al., 2007). In addition, the subgroup consisting of participants with polymorphic motivations includes only 24 individuals (14.9% of the total sample), which restricts the stability of parameter estimates (Wurpts & Geiser, 2014). Furthermore, the results of the between group comparison should be interpreted with caution, given the relatively modest sample size within this particular group. The size of this subgroup may also compromise the validity of the logistic regression model by influencing the size of the regression coefficients and/or the wideness of the confidence intervals (Horváth et al., 2023). Considering all these factors, it is also important to note that replication in larger samples will be necessary to confirm the robustness of the latent class structure. Nonetheless, it should be noted that the Multi NSSI functions class comprises more than 5% of the sample, which may help prevent overfitting the data and increase the likelihood of replication in other samples. In addition, LPA indices, including high entropy and LMRT, suggest that the class separation is statistically robust despite the small sample size of the group with highly polymorphic motivations. In the future, it would be worthwhile to test the validity of the NSSI severity factor identified in the present work on larger and representative samples to increase generalizability. Another limitation is the inability to fully test the differences between the two groups, as additional relevant variables—such as emotion regulation abilities and tolerance of uncertainty—not measured in the current study may also distinguish the groups. It is also worth noting that a community emerging adult sample was explored which may include students suffering from former or current mental illness. Ethical constraints prevented the assessment of psychiatric history, which could not subsequently be controlled. However, for instance, both emotional difficulties and NSSI constitute core symptoms in borderline personality disorder (Chen et al., 2023) and are closely associated with eating disorders (Kirkpatrick et al., 2023). Consequently, the severity of psychopathology and emotional dysregulation may influence both the likelihood of engaging in NSSI and the range of underlying motivations. Furthermore, individuals with more severe psychopathology often experience greater interpersonal difficulties (Girard et al., 2017), which may be particularly salient in a university setting and, in turn, contribute to the use of NSSI as a coping strategy. A further limitation was the gender imbalance in both the overall and NSSI subsamples, with males representing only one-quarter and one-fifth, respectively. Even though gender did not appear to influence NSSI function severity group membership, subsequent research should recruit representative samples to better assess possible differences in NSSI functionality between males and females.

## Conclusion

Both the Benefits and Barriers Model and the Two Functions Model, along with supporting empirical evidence, highlight that the greater the number of motivations underlying NSSI behavior, the more psychological problems are typically associated with it. However, to date, no study has employed latent class analysis to identify distinct subgroups derived solely from NSSI functions. This study used latent profile analysis to explore different patterns of several NSSI functions among emerging adult university students. According to a person-centered method, two robust classes emerged: a Multi NSSI functions subgroup, characterized by more impulsive engaging in NSSI for several intra- and interpersonal reasons, and an Only emotion regulation function subgroup who engaged in NSSI less intensely and primarily for emotion regulation purposes. The results underline the seriousness of NSSI engaging in several intrapersonal and interpersonal reasons. Thus, NSSI with multiple motivations is identified as a new NSSI severity indicator. Although a small proportion of undergraduates who engaged in NSSI in the past month belong to this vulnerable group, they can be characterized much poorer mental health than those who engaged in NSSI for “only” emotion regulation reasons. These results highlight the importance of considering this new indicator of NSSI severity in reducing self-harm, particularly in university settings. Identifying emerging adult undergraduates who engage in NSSI for multiple reasons is particularly justified given the alarming increase in NSSI prevalence during a transitive and complex period of development. The present findings contribute to identifying a group particularly vulnerable to psychopathology within higher education.

## Declarations

### Data Sharing Declaration

The data and the program code of the analyses that support the findings of this study are available at 10.17605/OSF.IO/P6NKM

## References

[CR1] Andreassen, C. S., Billieux, J., Griffiths, M. D., Kuss, D. J., Demetrovics, Z., Mazzoni, E., & Pallesen, S. (2016). The relationship between addictive use of social media and video games and symptoms of psychiatric disorders: A large-scale cross-sectional study. *Psychology of Addictive Behaviors*, *30*(2), 252–262. 10.1037/adb0000160. 26999354 10.1037/adb0000160

[CR2] Arnett, J. J. (2000). Emerging adulthood: A theory of development from the late teens through the twenties. *American Psychologist*, *55*(5), 469–480. 10.1037/0003-066X.55.5.469. 10842426

[CR3] Arnett, J. J. (2015). *Emerging adulthood: The winding road from the late teens through the twenties*. 2nd ed. Oxford University Press. 10.1093/oxfordhb/9780199795574.013.9

[CR4] Asparouhov T., Muthén B. (2014). *Auxiliary variables in mixture modeling: Using the BCH method in Mplus to estimate a distal outcome model and an arbitrary secondary model*. https://www.statmodel.com/download/asparouhov_muthen_2014.pdf

[CR5] Auerbach, R. P., Mortier, P., Bruffaerts, R., Alonso, J., Benjet, C., Cuijpers, P., Demyttenaere, K., Ebert, D. D., Green, J. G., Hasking, P., Murray, E., Nock, M. K., Pinder-Amaker, S., Sampson, N. A., Stein, D. J., Vilagut, G., Zaslavsky, A. M., & Kessler, R. C. (2018). WHO WMH-ICS Collaborators. WHO World Mental Health Surveys International College Student Project: Prevalence and distribution of mental disorders. *Journal of Abnormal Psychology*, *127*(7), 623–638. 10.1037/abn0000362. 30211576 10.1037/abn0000362 PMC6193834

[CR6] Bildik, T., Somer, O., Kabukcu Basay, B., Basay, O., & Ozbaran, B. (2013). The validity and reliability of the Turkish version of the Inventory of Statements About Self-injury. *Turkish Journal of Psychiatry*, *24*, 49–57. 10.5080/u6901. 23446540

[CR7] Boyne, H., & Hamza, C. A. (2022). Depressive symptoms, perceived stress, self-compassion and nonsuicidal self-injury among emerging adults: An examination of the between and within-person associations over time. *Emerging Adulthood*, *10*, 1269–1285. 10.1177/21676968211029768. 36111318 10.1177/21676968211029768 PMC9465554

[CR8] Bracken-Minor, K. L., McDevitt-Murphy, M. E., & Parra, G. R. (2012). Profiles of non-suicidal self-injurers and associated patterns of alcohol use. *Journal of Psychopathology and Behavioral Assessment*, *34*, 552–563. 10.1007/s10862-012-9306-5.

[CR9] Brackman, E. H., & Andover, A. S. (2017). Non-suicidal self-injury. In D. McKay, D.S. Abramowitz, & E.A. Storch (Eds.), *Treatments for Psychological Problems and Syndromes* (1st ed., 328–344). Wiley. ISBN-13:978-1118877005

[CR10] Brausch, A. M., & Muehlenkamp, J. J. (2018). Perceived effectiveness of NSSI in achieving functions on severity and suicide risk. *Psychiatry Research*, *265*, 144–150. 10.1016/j.psychres.2018.04.038. 29709788 10.1016/j.psychres.2018.04.038 PMC5984167

[CR11] Brown, R. C., & Plener, P. L. (2017). Non-suicidal self-injury in adolescence. *Current Psychiatry Reports*, *19*(3), 1–8. 10.1007/s11920-017-0767-9. 28315191 10.1007/s11920-017-0767-9 PMC5357256

[CR12] Brunner, R., Kaess, M., Parzer, P., Fischer, G., Carli, V., & Hoven, C. W., et al. (2014). Lifetime prevalence and psychosocial correlates of adolescent direct self-injurious behavior: A comparative study of findings in 11 European countries. *Journal of Child Psychology and Psychiatry*, *55*, 337–348. 10.1111/jcpp.12166. 24215434 10.1111/jcpp.12166

[CR13] Busa, F., Csima, M. P., Márton, J. A., Rozmann, N., Pandur, A. A., Ferkai, L. A., Deutsch, K., Kovács, Á., & Sipos, D. (2023). Sleep quality and perceived stress among health science students during online education. A single institution study. *Healthcare*, *12*, 75 10.3390/healthcare1201007. 38200981 10.3390/healthcare12010075 PMC10778774

[CR14] Calati, R., & Courtet, P. (2016). Is psychotherapy effective for reducing suicide attempt and non-suicidal self-injury rates? Meta-analysis and meta-regression of literature data. *Journal of Psychiatric Research*, *79*, 8–20. 10.1016/j.jpsychires.2016.04.003. 27128172 10.1016/j.jpsychires.2016.04.003

[CR15] Carey, M., Kerr-Gaffney, J., Strawbridge, R., Hieronymus, F., McCutcheon, R. A., Young, A. H., & Jauhar, S. (2025). Are cognitive behavioural therapy, cognitive therapy, and behavioural activation for depression effective in primary care? A systematic review and meta-analysis. *Journal of Affective Disorders*, *382*, 215–226. 10.1016/j.jad.2025.04.070. 40258424 10.1016/j.jad.2025.04.070

[CR16] Case, J. A. C., Burke, T. A., Siegel, D. M., Piccirillo, M. L., Alloy, L. B., & Olino, T. M. (2020). Functions of non-suicidal self-injury in late adolescence: A latent class analysis. *Archives of Suicide Research*, *24*(sup2), S165–S186. 10.1080/13811118.2019.1586607. 30856362 10.1080/13811118.2019.1586607 PMC6739178

[CR17] Chapman, A. L., Gratz, K. L., & Brown, M. Z. (2006). Solving the puzzle of deliberate self-harm: The experiential avoidance model. *Behaviour Research and Therapy*, *44*, 371–394. 10.1016/j.brat.2005.03.005. 16446150 10.1016/j.brat.2005.03.005

[CR18] Chen, Y., Fu, W., Ji, S., Zhang, W., Sun, L., Yang, T., He, K., & Zhou, Y. (2023). Relationship between borderline personality features, emotion regulation, and non-suicidal self-injury in depressed adolescents: a cross-sectional study. *BMC Psychiatry*, *23*, 293 10.1186/s12888-023-04800-1. 37118709 10.1186/s12888-023-04800-1 PMC10148398

[CR19] Cheng, C., Lau, Y. C., Chan, L., & Luk, J. W. (2021). Prevalence of social media addiction across 32 nations: meta-analysis with subgroup analysis of classification schemes and cultural values. *Addictive Behavior*, *117*, 106845 10.1016/j.addbeh.2021.106845. 10.1016/j.addbeh.2021.106845 33550200

[CR20] Chester, D. S., Merwin, L. M., & DeWall, C. N. (2015). Maladaptive perfectionism’s link to aggression and self-harm: Emotion regulation as a mechanism. *Aggressive Behavior*, *41*, 443–454. 10.1002/ab.21578. 26918433 10.1002/ab.21578

[CR21] Christoffersen, M. N., Møhl, B., DePanfilis, D., & Vammen, K. S. (2015). Non-suicidal self-injury–Does social support make a difference? An epidemiological investigation of a Danish national sample. *Child Abuse & Neglect*, *44*, 106–16. 10.1016/j.chiabu.2014.10.023. 25435107 10.1016/j.chiabu.2014.10.023

[CR22] Christoforou, R., Boyes, M., & Hasking, P. (2021). Emotion profiles of university students engaging in non-suicidal self-injury: Association with functions of self-injury and other mental health concerns. *Psychiatry Research*, *305*, 114253 10.1016/j.psychres.2021.114253. 34743063 10.1016/j.psychres.2021.114253

[CR23] Cipriano, A., Cella, S., & Cotrufo, P. (2017). Nonsuicidal self-injury: A systematic review. *Frontiers in Psychology*, *8*, 1–14. 10.3389/fpsyg.2017.01946. 29167651 10.3389/fpsyg.2017.01946 PMC5682335

[CR24] Clark, S. L., & Muthén, B. O. (2009). Relating latent class analysis results to variables not included in the analysis. Retrieved from http://www.statmodel.com/download/relatinglca.pdf

[CR25] Conley, C. S., Shapiro, J. B., Huguenel, B. M., & Kirsch, A. C. (2020). Navigating the college years: developmental trajectories and gender differences in psychological functioning, cognitive-affective strategies, and social well-being. *Emerging Adulthood*, *8*(2), 103–117. 10.1177/2167696818791603.

[CR26] da Silva Bandeira, B. E., Dos Santos Júnior, A., Dalgalarrondo, P., de Azevedo, R. C. S., & Celeri, E. H. V. R. (2022). Nonsuicidal self-injury in undergraduate students: A cross-sectional study and association with suicidal behavior. *Psychiatry Research*, *318*, 114917 10.1016/j.psychres.2022. 36332506 10.1016/j.psychres.2022.114917

[CR27] Dai, X., Zhang, Z., Sun, L., Zhu, S., Wu, Y., Lian, J., & Deng, X. (2025). Third-wave) cognitive behavioral therapy for generalized anxiety disorder in adults: A systematic review and Bayesian network meta-analysis. *Journal of Psychiatric Research*, *187*, 134–143. 10.1016/j.jpsychires.2025.05.010. 40367584 10.1016/j.jpsychires.2025.05.010

[CR28] Demetrovics, Z., Szeredi, B., & Rózsa, S. (2008). The three-factor model of Internet addiction: the development of the Problematic Internet Use Questionnaire. *Behavior Research Methods*, *40*(2), 563–574. 10.3758/brm.40.2.563. 18522068 10.3758/brm.40.2.563

[CR29] Farrell, B. C. T., Ewing, L., & Hamza, C. A. (2024). Examining trajectories of nonsuicidal self-injury across the first year of university. *Journal of Affective Disorders*, *15*(367), 202–209. 10.1016/j.jad.2024.09.003. 10.1016/j.jad.2024.09.003 39233238

[CR30] Girard, J. M., Wright, A. G. C., Beeney, J. E., Lazarus, S. A., Scott, L. N., Stepp, S. D., & Pilkonis, P. A. (2017). Interpersonal problems across levels of the psychopathology hierarchy. *Comprehensive Psychiatry*, *79*, 53–69. 10.1016/j.comppsych.2017.06.014. 28735709 10.1016/j.comppsych.2017.06.014 PMC5643217

[CR31] Gratz, K. L., Dixon-Gordon, K. L., Chapman, A. L., & Tull, M. T. (2015). Diagnosis and characterization of DSM-5 nonsuicidal self-injury disorder using the clinician-administered nonsuicidal self-injury disorder index. *Assessment*, *22*, 527–539. 10.1177/1073191114565878. 25604630 10.1177/1073191114565878 PMC5505727

[CR32] Guérin-Marion, C., Bureau, J. F., Lafontaine, M. F., Gaudreau, P., & Martin, J. (2021). Profiles of emotion dysregulation among university students who self-injure: Associations with parent-child relationships and non-suicidal self-injury characteristics. *Journal of Youth and Adolescence*, *50*, 767–787. 10.1007/s10964-020-01378-9. 33449284 10.1007/s10964-020-01378-9

[CR33] Guerry, J. D., & Prinstein, M. J. (2010). Longitudinal prediction of adolescent nonsuicidal self-injury: Examination of a cognitive vulnerability-stress model. *Journal of Clinical Child and Adolescent Psychology*, *39*, 77–89. 10.1080/15374410903401195. 20390800 10.1080/15374410903401195 PMC4626882

[CR34] Hamza, C. A., Stewart, S. L., & Willoughby, T. (2012). Examining the link between nonsuicidal self-injury and suicidal behavior: A review of the literature and an integrated model. *Clinical Psychology Review*, *32*(6), 482–495. 10.1016/j.cpr.2012.05.003. 22717336 10.1016/j.cpr.2012.05.003

[CR35] Hamza, C. A., Goldstein, A. L., Heath, N. L., & Ewing, L. (2021). Stressful experiences in university predict non-suicidal self-injury through emotional reactivity. *Frontiers in Psychology*, *12*, 610670 10.3389/fpsyg.2021.610670. 33927664 10.3389/fpsyg.2021.610670 PMC8076506

[CR36] Henry, J. D., & Crawford, J. R. (2005). The short-form version of the Depression Anxiety Stress Scales (DASS-21): Construct validity and normative data in a large non-clinical sample. *British Journal of Clinical Psychology*, *44*, 227–239. 10.1348/014466505X29657. 16004657 10.1348/014466505X29657

[CR37] Hooley, J. M., & Franklin, J. C. (2018). Why do people hurt themselves? A new conceptual model of nonsuicidal self-injury. *Clinical Psychological Science*, *6*(3), 428–451. 10.1177/2167702617745641.

[CR38] Horváth, Z., Paksi, B., Eisinger, A., Felvinczi, K., Demetrovics, O., & Demetrovics, Z. (2023). Longitudinal joint trajectories of gambling disorder and hypomentalization: A latent class growth analysis among young adults. *Comprehensive Psychiatry*, *126*, 152409 10.1016/j.comppsych.2023.152409. 37566950 10.1016/j.comppsych.2023.152409

[CR39] Jackson, K. M., Sher, K. J., & Park, A. (2005). Drinking among College Students. In M. Galanter (Ed.), *Recent Developments in Alcoholism: Vol. 17. Alcohol Problems in Adolescents and Young Adults* (pp. 85–117). Kluwer Academic/Plenum Publishers. 10.1007/0-306-48626-1_5 10.1007/0-306-48626-1_5 15789861

[CR40] Jarvi, S. M., Hearon, B. A., Batejan, K. L., Gironde, S., & Björgvinsson, T. (2017). Relations between past-week physical activity and recent nonsuicidal self-injury in treatment-seeking psychiatric adults. *Journal of Clinical Psychology*, *73*, 479–488. 10.1002/jclp.22342. 27391124 10.1002/jclp.22342

[CR41] Kelley, K. J., & Gruber, E. M. (2010). Psychometric properties of the Problematic Internet Use Questionnaire. *Computers in Human Behavior*, *26*, 1838–1845. 10.1016/j.chb.2010.07.018.

[CR42] Kia-Keating, M., No, U., Moore, S., Furlong, M. J., Liu, S., & You, S. (2018). Structural validity of the Depression, Anxiety, and Stress Scales-21 adapted for U.S. undergraduates. *Emerging Adulthood*, *6*(6), 434–440. 10.1177/2167696817745407.

[CR43] Kiekens, G., Hasking, P., Claes, L., Boyes, M., Mortier, P., Auerbach, R. P., Cuijpers, P., Demyttenaere, K., Green, J. G., Kessler, R. C., Myin-Germeys, I., Nock, M. K., & Bruffaerts, R. (2019). Predicting the incidence of non-suicidal self-injury in college students. *European Psychiatry*, *59*, 44–51. 10.1016/j.eurpsy.2019.04.002. 31035219 10.1016/j.eurpsy.2019.04.002

[CR44] Kiekens, G., Claes, L., Hasking, P., Mortier, P., Bootsma, E., Boyes, M., Myin-Germeys, I., Demyttenaere, K., Cuijpers, P., Kessler, R. C., Nock, M. K., & Bruffaerts, R. (2023). A longitudinal investigation of non-suicidal self-injury persistence patterns, risk factors, and clinical outcomes during the college period. *Psychological Medicine*, *53*(13), 6011–6026. 10.1017/S0033291722003178. 36325723 10.1017/S0033291722003178

[CR45] Kirkpatrick, R. H., Breton, E., Biorac, A., Munoz, D. P., & Booij, L. (2023). Non-suicidal self-injury among individuals with an eating disorder: A systematic review and prevalence meta-analysis. *International Journal of Eating Disorders*, *57*, 223–248. 10.1002/eat.24088. 38041221 10.1002/eat.24088

[CR46] Klonsky, E. D. (2007). The functions of deliberate self-injury: A review of the evidence. *Clinical Psychology Review*, *27*, 226–239. 10.1016/j.cpr.2006.08.002. 17014942 10.1016/j.cpr.2006.08.002

[CR47] Klonsky, E. D. (2011). Non-suicidal self-injury in United States adults: prevalence, sociodemographics, topography and functions. *Psychological Medicine*, *41*(9), 1981–1986. 10.1017/S0033291710002497. 21208494 10.1017/S0033291710002497

[CR48] Klonsky, E. D., & Olino, T. M. (2008). Identifying clinically distinct subgroups of self-injurers among young adults: A latent class analysis. *Journal of Consulting and Clinical Psychology*, *76*, 22–27. 10.1037/0022-006X.76.1.22. 18229979 10.1037/0022-006X.76.1.22

[CR49] Klonsky, E. D., & Glenn, C. R. (2009). Assessing the functions of non-suicidal self-injury: Psychometric properties of the Inventory of Statements About Self-Injury (ISAS). *Journal of Psychopathology and Behavioral Assessment*, *31*, 215–219. 10.1007/s10862-008-9107-z. 29269992 10.1007/s10862-008-9107-z PMC5736316

[CR50] Klonksy, E. D., Glenn, C. R., Styer, D. M., Olino, T. M., & Washburn, J. J. (2015). The functions of nonsuicidal self-injury: Converging evidence for a two-factor structure. *Child and Adolescent Psychiatry and Mental Health*, *9*, 44 10.1186/s13034-015-0073-4. 26421059 10.1186/s13034-015-0073-4 PMC4586000

[CR51] La Guardia, A. C., Cramer, R. J., Bryson, C. N., & Emelianchik‐Key, K. (2020). Analysis of personality, suicide, and self‐injury in emerging adulthood. *Journal of College Counseling*, *23*(1), 57–70. 10.1002/jocc.12149.

[CR52] Linehan, M. M., Korslund, K. E., Harned, M. S., Gallop, R. J., Lungu, A., Neacsiu, A. D., McDavid, J., Comtois, K. A., & Murray-Gregory, A. M. (2015). Dialectical behavior therapy for high suicide risk in individuals with borderline personality disorder: A randomized clinical trial and component analysis. *JAMA Psychiatry*, *72*, 475–482. 10.1001/jamapsychiatry.2014.3039. 25806661 10.1001/jamapsychiatry.2014.3039

[CR53] Liu, R. T. (2023). The epidemiology of non-suicidal self-injury: lifetime prevalence, sociodemographic and clinical correlates, and treatment use in a nationally representative sample of adults in England. *Psychological Medicine*, *53*(1), 274–282. 10.1017/S003329172100146X. 33960286 10.1017/S003329172100146X PMC10324294

[CR54] Liu, R. T., Cheek, S. M., & Nestor, B. A. (2016). Non-suicidal self-injury and life stress: A systematic meta-analysis and theoretical elaboration. *Clinical Psychology Review*, *47*, 1–14. 10.1016/j.cpr.2016.05.005. 27267345 10.1016/j.cpr.2016.05.005 PMC4938721

[CR55] Liu, X., Gui, Z., Chen, Z. M., Feng, Y., Wu, X. D., Su, Z., Cheung, T., Ungvari, G. S., Liu, X. C., Yan, Y. R., Ng, C. H., & Xiang, Y. T. (2025). Global prevalence of internet addiction among university students: a systematic review and meta-analysis. *Current Opinion in Psychiatry*, *38*, 182–199. 10.1097/YCO.0000000000000994. 40009750 10.1097/YCO.0000000000000994

[CR56] Lloyd-Richardson, E. E., Perrine, N., Dierker, L., & Kelley, M. L. (2007). Characteristics and functions of non-suicidal self-injury in a community sample of adolescents. *Psychological Medicine*, *37*(8), 1183–1192. 10.1017/S003329170700027X. 17349105 10.1017/S003329170700027X PMC2538378

[CR57] Lovibond, S. H., & Lovibond, P. F. (1995). *Manual for the Depression Anxiety & Stress Scales*. 2nd Ed. Psychology Foundation. http://www2.psy.unsw.edu.au/DASS/

[CR58] Mannekote Thippaiah, S., Shankarapura Nanjappa, M., & Gude, J. G. (2021). Non-suicidal self-injury in developing countries: A review. *International Journal of Social Psychiatry*, *67*(5), 472–482. 10.1177/0020764020943627. 32715834 10.1177/0020764020943627

[CR59] Miller, A. E., & Racine, S. E. (2022). Emotion regulation difficulties as common and unique predictors of impulsive behaviors in university students. *Journal of American College Health*, *70*(5), 1387–1395. 10.1080/07448481.2020.1799804. 32790500 10.1080/07448481.2020.1799804

[CR60] Møhl, B. (2020). *Assessment and treatmnet of non-suicidal self-injury. A clinical perspective*. (Routledge. ISBN-13:978-1138349803.

[CR61] Muthén, L. K., & Muthén, B. O. (2007). *Mplus user guide*. 5th ed. Muthén & Muthén. 1998–.

[CR62] Nock, M. K. (2009). Why do people hurt themselves? New insights into the nature and functions of self-injury. *Current Directions in Psychological Science*, *18*, 78–83. 10.1111/j.1467-8721.2009.01613.x. 20161092 10.1111/j.1467-8721.2009.01613.x PMC2744421

[CR63] Nock, M. K. (2010). Self-injury. *Annual Review of Clinical Psychology*, *6*, 339–363. 10.1146/annurev.clinpsy.121208.131258. 20192787 10.1146/annurev.clinpsy.121208.131258

[CR64] Nock, M. K., & Prinstein, M. J. (2004). A functional approach to the assessment of self-mutilative behavior. *Journal of Consulting and Clinical Psychology*, *72*, 885–890. 10.1037/0022-006X.72.5.885. 15482046 10.1037/0022-006X.72.5.885

[CR65] Nylund, K. L., Asparouhov, T., & Muthen, B. O. (2007). Deciding on the number of classes in latent class analysis and growth mixture modeling: A Monte Carlo simulation study. *Structural Equation Modeling: A Multidisciplinary Journal*, *14*, 535–569. 10.1080/10705510701575396.

[CR66] Paiva, U., Cortese, S., Flor, M., Moncada-Parra, A., Lecumberri, A., Eudave, L., Magallón, S., García-González, S., Sobrino-Morras, Á., Piqué, I., Mestre-Bach, G., Solmi, M., & Arrondo, G. (2025). Prevalence of mental disorder symptoms among university students: An umbrella review. *Neuroscience and Biobehavioral Review*, *175*, 106244 10.1016/j.neubiorev.2025.106244. 10.1016/j.neubiorev.2025.106244 40480638

[CR67] Park, H. (2025). The relationship between career stress and non-suicidal self-injury among college students: Mediating effects of depression and resilience. *Acta Psychologica*, *257*, 105078 10.1016/j.actpsy.2025.105078. 40378610 10.1016/j.actpsy.2025.105078

[CR68] Pedrelli, P., Nyer, M., Yeung, A., Zulauf, C., & Wilens, T. (2015). College students: mental health problems and treatment considerations. *Academic Psychiatry*, *39*(5), 503–511. 10.1007/s40596-014-0205-9. 25142250 10.1007/s40596-014-0205-9 PMC4527955

[CR69] Pérez, S., García-Alandete, J., Cañabate, M., & Marco, J. H. (2020). Confirmatory factor analysis of the Inventory of Statement About Self-injury in a Spanish clinical sample. *Journal of Clinical Psychology*, *76*, 102–117. 10.1002/jclp.22844. 31454078 10.1002/jclp.22844

[CR70] Peterson, A. L., Chen, J. I., Karver, M. S., & Labouliere, C. D. (2019). Frustration with feeling: Latent classes of non-suicidal self-injury and emotion regulation difficulties. *Psychiatry Research*, *275*, 61–70. 10.1016/j.psychres.2019.03.014. 30878858 10.1016/j.psychres.2019.03.014 PMC6543814

[CR71] Qu, D., Wen, X., Liu, B., Zhang, X., He, Y., Chen, D., Duan, X., Yu, J., Liu, D., Zhang, X., Ou, J., Zhou, J., Cui, Z., An, J., Wang, Y., Zhou, X., Yuan, T., Tang, J., Yue, W., & Chen, R. (2023). Non-suicidal self-injury in Chinese population: A scoping review of prevalence, method, risk factors and preventive interventions. *Lancet Regional Health Western Pacific*, *37*, 100794 10.1016/j.lanwpc.2023.100794. 37693882 10.1016/j.lanwpc.2023.100794 PMC10485683

[CR72] Rivers, S. E., Brackett, M. A., Omori, M., Sickler, C., Bertoli, M. C., & Salovey, P. (2013). Emotion skills as a protective factor for risky behaviors among college students. *Journal of College Student Development*, *54*(2), 172–183. 10.1353/csd.2013.0012.

[CR73] Salari, N., Zarei, H., Hosseinian-Far, A., Rasoulpoor, S., Shohaimi, S., & Mohammadi, M. (2025). The global prevalence of social media addiction among university students: A systematic review and meta-analysis. *Journal of Public Health*, *33*(1), 223–236. 10.1007/s10389-023-02012-1.

[CR74] Saunders, J. B., Aasland, O. G., Babor, T. F., de la Fuente, J. R., & Grant, M. (1993). Development of the alcohol use disorders identification test (AUDIT): WHO collaborative project on early detection of persons with harmful alcohol consumption–II. *Addiction*, *88*(6), 791–804. 10.1111/j.1360-0443.1993.tb02093.x. 8329970 10.1111/j.1360-0443.1993.tb02093.x

[CR75] Sawyer, S. M., Azzopardi, P. S., Wickremarathne, D., & Patton, G. C. (2018). The age of adolescence. *The Lancet Child & Adolescent Health*, *2*(3), 223–228. 10.1016/S2352-4642(18)30022-1. 30169257 10.1016/S2352-4642(18)30022-1

[CR76] Schmidt, C., Nicolaou, S., Pascual, J. C., Puntí, J., Lara, A., Sintes, A., Méndez, I., Romero, S., Briones-Buixassa, L., Santamarina-Perez, P., Soler, J., & Vega, D. (2024). Identifying high-risk subgroups of college students with non-suicidal self-injury: A latent profile analysis and two-years follow-up study. *Journal of Youth and Adolescence*, *53*, 1370–1382. 10.1007/s10964-024-01970-3. 38553580 10.1007/s10964-024-01970-3

[CR77] Serras, A., Saules, K. K., Cranford, J. A., & Eisenberg, D. (2010). Self-injury, substance use, and associated risk factors in a multi-campus probability sample of college students. *Psychology of Addictive Behaviors*, *24*(1), 119–128. 10.1037/a0017210. 20307119 10.1037/a0017210

[CR78] Shin, N. Y. (2022). Psychometric properties of the Bergen Social Media Addiction Scale in Korean young adults. *Psychiatry Investigation*, *19*, 356–361. 10.30773/pi.2021.0294. 35620820 10.30773/pi.2021.0294 PMC9136528

[CR79] Singhal, N., Bhola, P., Reddi, V. S. K., Bhaskarapillai, B., & Joseph, S. (2021). Non-suicidal self-injury (NSSI) among emerging adults: Sub-group profiles and their clinical relevance. *Psychiatry Research*, *300*, 113877 10.1016/j.psychres.2021.113877. 33831810 10.1016/j.psychres.2021.113877

[CR80] Skogen, J. C., Thørrisen, M. M., Knudsen, A. K. S., Reneflot, A., & Sivertsen, B. (2024). Screening student drinking behaviors: Examining AUDIT criterion validity using CIDI-based alcohol use disorder as the ‘gold standard’. *Frontiers in Public Health*, *12*, 1328819 10.3389/fpubh.2024.1328819. 38737856 10.3389/fpubh.2024.1328819 PMC11082383

[CR81] Somer, O., Bildik, T., Kabukçu-Başay, B., Güngür, D., Başay, Ö., & Farmer, R. F. (2015). Prevalence of non-suicidal self-injury and distinct groups of self-injurers in a community sample of adolescents. *Social Psychiatry and Psychiatric Epidemiology*, *50*, 1163–1171. 10.1007/s00127-015-1060-z. 25952581 10.1007/s00127-015-1060-z

[CR82] Soldatos, C. R., Dikeos, D. G., & Paparrigopoulos, T. J. (2000). Athens Insomnia Scale: validation of an instrument based on ICD-10 criteria. *Journal of Psychosomatic Research*, *48*(6), 555–560. 10.1016/s0022-3999(00)00095-7. 11033374 10.1016/s0022-3999(00)00095-7

[CR83] Steinberg, L. (2008). A social neuroscience perspective on adolescent risk-taking. *Developmental Review*, *28*(1), 78–106. 10.1016/j.dr.2007.08.002. 18509515 10.1016/j.dr.2007.08.002 PMC2396566

[CR84] St. Germain, S. A., & Hooley, J. M. (2012). Direct and indirect forms of non-suicidal self-injury: Evidence for a distinction. *Psychiatry Research*, *197*, 78–84. 10.1016/j.psychres.2011.12.050. 22406394 10.1016/j.psychres.2011.12.050

[CR85] Taylor, P. J., Jomar, K., Dhingra, K., Forrester, R., & Shahmalak, U. (2018). A meta-analysis of the prevalence of different functions of non-suicidal self-injury. *Journal of Affective Disorders*, *227*, 759–769. 10.1016/j.jad.2017.11.073. 29689691 10.1016/j.jad.2017.11.073

[CR86] Uliaszek, A. A., Rashid, T., Williams, G. E., & Gulamani, T. (2016). Group therapy for university students: A randomized control trial of dialectical behavior therapy and positive psychotherapy. *Behaviour Research and Therapy*, *77*, 78–85. 10.1016/j.brat.2015.12.003. 26731172 10.1016/j.brat.2015.12.003

[CR87] Victor, S. E., Styer, D., & Washburn, J. J. (2016). Functions of nonsuicidal self-injury (NSSI): cross-sectional associations with NSSI duration and longitudinal changes over time and following treatment. *Psychiatry Research*, *241*, 83–90. 10.1016/j.psychres.2016.04.083. 27156029 10.1016/j.psychres.2016.04.083

[CR88] Voon, D., Hasking, P., & Martin, G. (2014). The roles of emotion regulation and ruminative thoughts in non-suicidal self-injury. *British Journal of Clinical Psychology*, *53*, 95–113. 10.1111/bjc.12030. 24117940 10.1111/bjc.12030

[CR89] Wang, Y. J., Li, X., Ng, C. H., Xu, D. W., Hu, S., & Yuan, T. F. (2022). Risk factors for non-suicidal self-injury (NSSI) in adolescents: a meta-analysis. *eClinicalMedicine*, *46*, 101350 10.1016/j.eclinm.2022.101350. 35330803 10.1016/j.eclinm.2022.101350 PMC8938878

[CR90] Wang, Z., Li, D., Chen, Y., Tao, Z., Jiang, L., He, X., & Zhang, W. (2024). Understanding the subtypes of non-suicidal self-injury: A new conceptual framework based on a systematic review. *Psychiatry Research*, *334*, 115816 10.1016/j.psychres.2024.115816. 38412712 10.1016/j.psychres.2024.115816

[CR91] Washburn, J. J., Klonsky, E. D., Styer, D. M., Gebhardt, M., Juzwin, K. R., & Aldridge, D. (2012). Short form of the Inventory of Statements About Self-Injury. Poster presentation at the 7^th^ Annual Meeting of the International Society for the Study of Self-Injury. Chapel Hill, NC.

[CR92] Wester, K., Trepal, H., & King, K. (2018). Nonsuicidal self-injury: Increased prevalence in engagement. *Suicide and Life-Threatening Behavior*, *48*, 690–698. 10.1111/sltb.12389. 28846813 10.1111/sltb.12389

[CR93] Whitlock, J., Eckenrode, J., & Silverman, D. (2006). Self-injurious behaviors in a college population. *Pediatrics*, *117*(6), 1939–1948. 10.1542/peds.2005-2543. 16740834 10.1542/peds.2005-2543

[CR94] World Medical Association. (2013). World Medical Association Declaration of Helsinki: Ethical principles for medical research involving human subjects. *JAMA*, *310*, 2191–2194. 10.1001/jama.2013.281053. 24141714 10.1001/jama.2013.281053

[CR95] Wurpts, I. C., & Geiser, C. (2014). Is adding more indicators to a latent class analysis beneficial or detrimental? Results of a Monte-Carlo study. *Frontiers in Psychology*, *5*, 920 10.3389/fpsyg.2014.00920. 25191298 10.3389/fpsyg.2014.00920 PMC4140387

